# Feasibility study of electrocardiographic and respiratory gated, gadolinium enhanced magnetic resonance angiography of pulmonary veins and the impact of heart rate and rhythm on study quality

**DOI:** 10.1186/1532-429X-16-43

**Published:** 2014-06-19

**Authors:** John D Groarke, Alfonso H Waller, Tomas S Vita, Gregory F Michaud, Marcelo F Di Carli, Ron Blankstein, Raymond Y Kwong, Michael Steigner

**Affiliations:** 1 Cardiovascular Imaging Program, Cardiovascular Division, Department of Medicine and Department of Radiology, Brigham and Women’s Hospital, Harvard Medical School, Boston, MA, USA; 2 Cardiovascular Division, Department of Medicine, Brigham and Women’s Hospital, Harvard Medical School, Boston, MA, USA

**Keywords:** Pulmonary vein imaging, Respiratory gated, ECG gated, Magnetic resonance angiography, 3 Tesla, Image segmentation, Electroanatomic mapping systems, Pre ablation imaging, End-systole

## Abstract

**Background:**

We aimed to assess the feasibility of 3 dimensional (3D) respiratory and ECG gated, gadolinium enhanced magnetic resonance angiography (MRA) on a 3 Tesla (3 T) scanner for imaging pulmonary veins (PV) and left atrium (LA). The impact of heart rate (HR) and rhythm irregularity associated with atrial fibrillation (AF) on image and segmentation qualities were also assessed.

**Methods:**

101 consecutive patients underwent respiratory and ECG gated (ventricular end systolic window) MRA for pre AF ablation imaging. Image quality (assessed by PV delineation) was scored as 1 = not visualized, 2 = poor, 3 = good and 4 = excellent. Segmentation quality was scored on a similar 4 point scale. Signal to noise ratios (SNRs) were calculated for the LA, LA appendage (LAA), and PV. Contrast to noise ratios (CNRs) were calculated between myocardium and LA, LAA and PV, respectively. Associations between HR/rhythm and quality metrics were assessed.

**Results:**

35 of 101 (34.7%) patients were in AF at time of MRA. 100 (99%) patients had diagnostic studies, and 91 (90.1%) were of good or excellent quality. Overall, mean ± standard deviation (SD) image quality score was 3.40 ± 0.69. Inter observer agreement for image quality scores was substantial, (kappa = 0.68; 95% confidence interval (CI): 0.46, 0.90). Neither HR adjusting for rhythm [odds ratio (OR) = 1.03, 95% CI = 0.98,1.09; p = 0.22] nor rhythm adjusting for HR [OR = 1.25, 95% CI = 0.20, 7.69; p = 0.81] demonstrated association with image quality. Similarly, SNRs and CNRs were largely independent of HR after adjusting for rhythm. Segmentation quality scores were good or excellent for 77.3% of patients: mean ± SD score = 2.91 ± 0.63, and scores did not significantly differ by baseline rhythm (p = 0.78).

**Conclusions:**

3D respiratory and ECG gated, gadolinium enhanced MRA of the PVs and LA on a 3 T system is feasible during ventricular end systole, achieving high image quality and high quality image segmentation when imported into electroanatomic mapping systems. Quality is independent of HR and heart rhythm for this free breathing, radiation free, alternative strategy to current MRA or CT based approaches, for pre AF ablation imaging of PVs and LA.

## Background

Catheter ablation of atrial fibrillation (AF) is considered appropriate treatment for symptomatic AF, refractory or intolerant to at least one antiarrhythmic medication, and may be considered as first line treatment for certain patients [1]. The rate of catheter ablation in patients with AF, across all age groups, is increasing significantly over time [2]. Computerized tomography (CT) or cardiovascular magnetic resonance (CMR) evaluation of the left atrium (LA) and pulmonary vein (PV) anatomy prior to catheter ablation is considered appropriate [3]. Such imaging provides accurate visualization of highly variable PV and LA anatomy, facilitates image integration with electroanatomic mapping systems, and demonstrates the atrioesophageal relationship that is important for risk assessment of thermal esophageal injury. Integration of pre-acquired cardiac images with electroanatomic mapping to guide catheter ablations is feasible and inconsistently reported to improve procedural success, reduce procedure duration, fluoroscopy time and occurrence of PV stenosis, compared to conventional electroanatomic mapping alone [4-10]. For example, PV isolation guided by image integration was associated with reduced AF recurrence in comparison with PV isolation guided by three dimensional (3D) electroanatomical mapping alone based on registry data from 573 patients undergoing catheter ablation for paroxysmal AF [10]; however, randomized trials of AF ablation guided by 3D electroanatomical mapping alone versus with image integration have shown no difference in AF outcomes [8,9].

CT angiography (CTA) of the PVs and LA offers high spatial resolution and fast acquisition times. However, CTA requires the use of iodinated contrast agents and radiation exposure, which increases overall radiation exposure when added to fluoroscopy related exposure during catheter ablation. Therefore, for patients with no contraindication, CMR is increasingly preferred for pre ablation PV and LA imaging. There is no significant difference in registration accuracy during image integration into electroanatomic mapping systems with contrast enhanced CT imaging versus gadolinium enhanced CMR [4,11]. CMR sequences for PV and LA imaging without intravenous contrast agents are used clinically; however, contrast enhanced CT is reported to provide superior LA anatomy reconstruction compared to a non contrast CMR dataset [12]. Similarly, non-contrast CMR sequences have been shown to be of significantly inferior quality compared to contrast enhanced MR images [13,14]. In clinical practice, CMR of the PVs is most often performed by contrast enhanced MR angiography (MRA) during an expiratory breath-hold, without electrocardiographic (ECG) gating that would prolong breath-hold time. Free breathing, respiratory and ECG gated MRA of the LA and PVs may offer higher spatial resolution and less motion artifacts through ECG gating than the conventional breath held MRA [14]. Furthermore, accurate registration during image integration into electroanatomical mapping systems is critical [1]. Patients are free breathing throughout catheter ablation procedures; respiratory related changes in LA and PV anatomy during breath held imaging techniques may predispose to registration errors during image integration [15]. Free breathing imaging techniques may be preferable to either breath hold MRA or CT techniques, resembling the breathing pattern during electroanatomic mapping at catheter ablation.

Small studies have demonstrated the feasibility of free breathing, respiratory gated CMR for LA and PV imaging, with and without contrast enhancement [14,16-19]. The impact of heart rate (HR) and rhythm on image quality and registration accuracy, outside of data from very small studies, are uncertain [20,21]. End-systolic imaging has been suggested to improve image quality during magnetic resonance coronary angiography compared to diastolic acquisitions in 14 subjects with heart rates exceeding 65 beats/minute using a 1.5 tesla (T) MR scanner [22], and among 10 volunteers imaged using a 3 T scanner [23]. However, the feasibility of ECG triggering at ventricular *end-systole*, the phase of the cardiac cycle least sensitive to increases in heart rate [24] and irregular rhythm, has not yet been described in a large number of patients undergoing MRA with variable heart rhythm and heart rates.

The purpose of this study was to determine the feasibility and diagnostic quality of free breathing, respiratory and end-systolic ECG gated, contrast enhanced MRA of the PV and LA anatomy on a 3 T scanner in an unselected cohort of consecutive patients referred for pre-ablation imaging. The impact of HR and rhythm at the time of image acquisition on image quality, and the quality of image segmentation obtained from these 3D MRA datasets using image integration software of an electroanatomic mapping system require investigation.

## Methods

### Study population

101 consecutive patients referred for pre-catheter ablation MR imaging of the PVs and LA over an eight month study period were included in this prospective study. The study was approved by the Institutional Review Board and was compliant with the Health Insurance Portability and Accountability Act. The requirement for informed consent was waived because of the nature of the study. Baseline demographics, including HR and rhythm, were recorded for all patients at the time of CMR.

### Image acquisition

**Figure 1:**
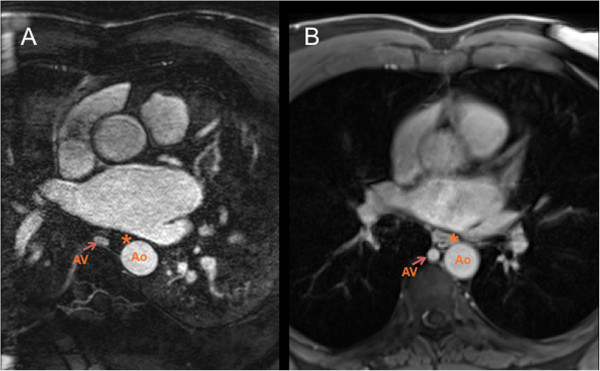
**Anatomical relationship of the pulmonary veins to the esophagus. A**- Axial slice from a 3D respiratory- and ECG-gated gadolinium-enhanced MRA demonstrating that the esophagus (labeled *) can be difficult to identify on this sequence. **B**- Axial T1- weighted, fat-saturated 3D-gradient echo sequence from the same patient at a similar level clearly demonstrating the esophagus and its anatomical relations. [Key: AV = azygos vein, Ao = descending thoracic aorta, * = esophagus].

All patients were scanned on a commercial 3 T MRI scanner (TimTrio, Siemens, Erlangen, Germany). Electrocardiographic electrodes were positioned for optimal gating before the study. Conventional multiplanar scout images were obtained followed by a high resolution four chamber steady state free precession (SSFP) sequence (echo time : 1 ms, repetition time (TR): 24.24 ms; flip angle : 50 degree; field of view: 340 mm; readout bandwidth: 930 Hz/pixel; matrix size: 1.8 x 1.3 x 8.0 mm; slice thickness: 8 mm; parallel imaging with an acceleration factor of 3 was applied) was used to identify the cardiac phase corresponding to ***end-systole*** by visual assessment. The timing of this end-systolic phase was then entered as the trigger delay for acquisitions. The acquisition window duration was set at 100 ms for all patients, given that the end-systolic phase that lasts approximately 100–150 ms is more or less independent of heart rate and related RR interval [25]. Free breathing, contrast enhanced MRA using a ECG triggered, respiratory gated, inversion recovery prepared, 3D volume whole heart acquisition segmented gradient echo sequence (echo time: 1.4 ms, echo spacing: 3.22 ms, number of k space lines per cardiac cycle: 35, flip angle: 20 degrees, inversion time = 200 ms, readout bandwidth = 698 Hz/pixel, slice thickness: 1.2 mm, acquired voxel size: 1.3 × 1.3 × 1.2 mm3, 88–120 slices) was obtained, starting 45 seconds after initiation of an intravenous infusion of 0.15 mmol/kg of gadobenate dimeglumine (MultiHance®; Bracco Imaging SpA, Milan, Italy) at a rate of 0.3 ml/sec using a power injector (MEDRAD Inc., Warrendale, PA, USA), followed by 20 ml saline at the same rate. Gadolinium dimeglumine with a higher T1 relaxivity relative to other gadolinium chelates [26], a dosing regimen similar to that used in a smaller study [16], and the infusion protocol were all selected in an effort to maintain maximum blood pool enhancement for the duration of MRA acquisition. The FOV was selected to include the entire LA, LA appendage (LAA) and proximal PVs. An axial slab and a coronal image taken at end-expiration were used to position the navigator beam on the dome of the right hemi diaphragm to track the end expiratory position of the diaphragm to achieve respiratory gating, with an acceptance window of 5 mm. The position of the navigator bands was adjusted on an axial image to a location lateral to the proximal right pulmonary veins prior to acquisition. A parallel imaging technique with an acceleration factor of two was used to shorten acquisition times. An axial T1 weighted, fat saturated 3D gradient echo sequence (echo time: 1.26 ms, TR: 3.51 ms, flip angle: 10 degrees, readout bandwidth = 500 Hz/pixel; slice thickness: 4.0 mm) was performed after completion of the MRA to demonstrate the course of the esophagus relative to the pulmonary veins (Figure 1).

### Image analysis

#### Qualitative analysis

**Figure 2:**
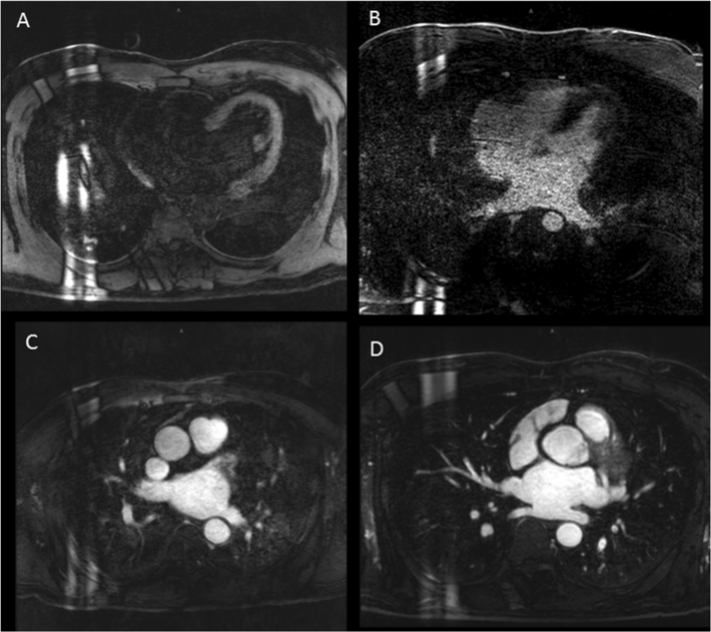
**Qualitative analysis of image quality. A**- Image quality grade 1: pulmonary veins not visualized; **B**- Grade 2: pulmonary veins poorly defined with significant blurring of vessels; **C**- Grade 3: pulmonary veins well defined with mild blurring of vessels; **D**- Grade 4: excellent pulmonary vein definition without blurring.

Image quality, as assessed by visibility and definition of pulmonary veins on the 3D volume acquisition dataset from each study, was graded on a four point scale (Figure 2), similar to scales used in other studies [17,27]: 1: not visualized; 2: poorly defined with blurring such that stenosis or diameter could not be confidently evaluated; 3: well defined with mild blurring only; 4: excellent image quality without blurring. These analyses were performed by two readers blinded to each other, and to HR and rhythm at time of imaging. A third blinded reader scored cases where quality scores were discordant (n = 26). As such, a consensus quality score was determined for all cases, and these scores were used for quality scores included in analyses.

#### Quantitative analysis

The following data were calculated for each subject using QMass Enterprise Solution 7.4® (Medis Medical Imaging System, Inc., Leiden, Netherlands); formulae used were similar to those used in published studies [14,28]:

A) Signal to noise ratios (SNRs):

A region of interest (ROI) with an area of at least 1.0 cm^2^ was placed in the LA and the mean signal intensity (SI) and standard deviation (SD) was calculated. The SNR_LA_ is calculated using the following formula: (mean SI in blood in LA/SD). A similar method using a ROI within the largest PV and within the LAA was used to calculate the SNR_PV_ and SNR_LAA_, respectively.

B) Contrast to noise ratios (CNRs):

A ROI with an area of at least 1.0 cm^2^ was placed in the LA and also in the myocardium (basal anteroseptum) and the mean SI and SD for each site was calculated. The CNR_LA/myocardium_ was calculated using the following formula: (mean SI in blood - mean SI in myocardium)/(0.5 x [SD in blood + SD in myocardium]). A similar method using a ROI within the largest PV and within the LAA was used to calculate CNR_PV/myocardium_ and CNR_LAA/myocardium_, respectively.

### Image segmentation analyses

**Figure 3:**
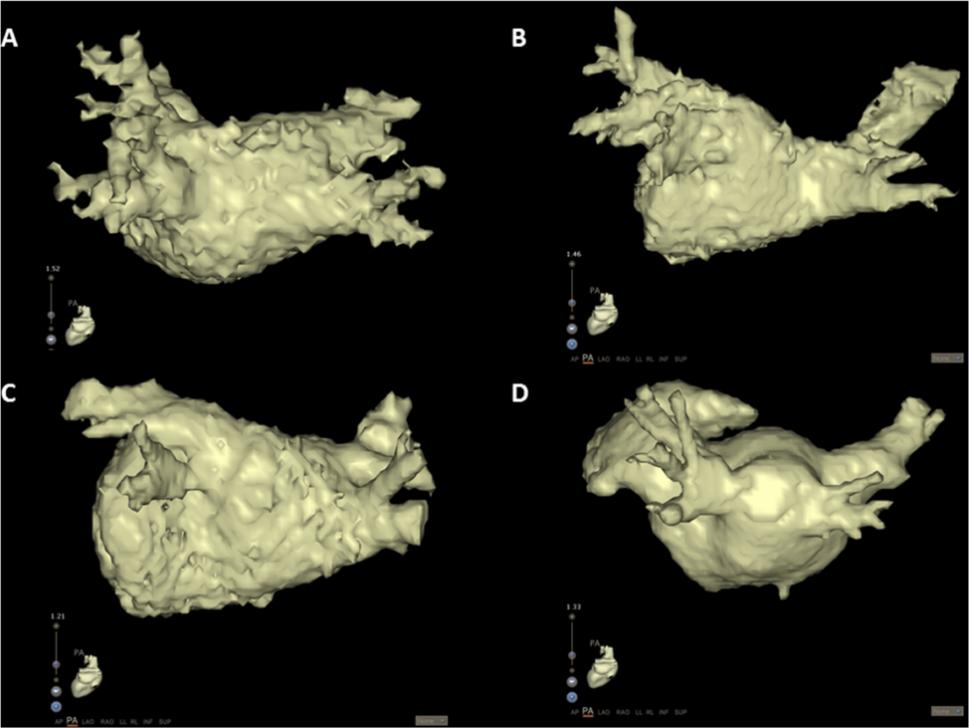
Image segmentation scoring: A- Grade 1: poor segmentation due to inability to separate LA and PVs from adjacent structures; B- Grade 2: moderate segmentation with incomplete separation of LA and PVs from adjacent structures; C- Grade 3: good segmentation with near complete separation of LA and PVs; D- excellent segmentation with complete separation of LA and PV.

MRA datasets were imported into an electroanatomic mapping system (CartoMerge Image Integration Module, Biosense Webster Inc., Diamond Bar, CA USA). Using CartoMerge semi automated image integration software, a technician with over 10 years of experience, blinded to other results, segmented the LA and PVs. Segmentation quality was then assessed according to a 4 point score, similar to that used by Wagner et al. [14]: 1- poor segmentation quality due to inability to separate LA and PVs from adjacent structures; 2- moderate segmentation quality with incomplete separation of LA and PVs from adjacent structures; 3- good segmentation quality with near complete separation of LA and PVs, and 4- excellent segmentation quality with complete separation of LA and PVs achieved (Figure 3).

### Patient follow up

Complications related to catheter ablation of AF were defined as per consensus guidelines [29] and recorded with interval from procedure for all patients. Patients were followed with clinic evaluations, 12 lead electrocardiogram, and/or 24 hour Holter monitor to detect recurrent atrial arrhythmias. Atrial arrhythmias (including documented AF, atrial flutter, or atrial tachycardias) after a 3 month blanking period following ablation were defined as recurrences.

### Statistical analyses

Continuous, normally distributed data are presented as mean ± SD. Continuous, non-normal data are presented as median with interquartile range (IQR). Categorical data are presented as percentages. Data are presented for entire patient cohort, patients in normal sinus rhythm (NSR) and patients in AF at the time of imaging. Continuous variables and binary variables are compared between NSR and AF patient cohorts using a two tailed Student’s t test and Fisher’s exact test, respectively. The Mantel Haenszel Chi Square test is used to test for group comparisons of image quality and segmentation quality scores. The image quality scores of readers 1 and 2 are compared using a paired t test, dichotomized as poor (quality score =1 or 2) or good (quality score = 3 or 4), and inter observer agreement for dichotomized quality scores between readers is determined by calculating the kappa statistic.

The crude relationship between dichotomized consensus image quality (score ≥3 good versus score ≤2 poor) and heart rhythm is presented as an unadjusted odds ratio (OR), with associated 95% confidence intervals (CI). To adjust for the effect of HR at the time of CMR, a logistic regression model with dichotomized consensus image quality as the dependent variable and HR and heart rhythm at time of CMR as predictor variables is used. Effect modification by HR on the association between heart rhythm and image quality is investigated using the same model with the inclusion of an interaction variable. To assess for non linear associations with HR, HR was tested in four formats: (i) continuous linear relationship, (ii) categorical relationship [3 categories: HR ≤ 55 bpm (n = 21), 55 < HR ≤ 90 bpm (n = 66), and HR > 90 bpm (n = 14)], (iii) log transformation of HR, and (iv) quadratic relationship. Multiple linear regression models were used to assess the relationship between categorical HR and rhythm with each of the SNRs and CNRs. A p value of <0.05 was considered significant. Analyses were performed using SAS 9.3® (SAS Institute Inc., Cary, NC, USA).

## Results

### Patient characteristics

101 patients were included in this study. All patients had a history of persistent or paroxysmal AF. 35 (34.7%) patients were in AF at the time of CMR. Baseline characteristics for entire patient cohort, as well as by AF status at time of imaging are outlined in Table 1. Patients in AF at time of CMR had a significantly higher mean HR and lower mean systolic BP (SBP) at the time of imaging, lower mean left ventricular ejection fraction (LVEF) and larger mean LA diameter, as measured on a 3 chamber SSFP sequence at end-systole, than patients in NSR.

**Table 1:** Baseline characteristics presented by entire cohort and by AF status

	**Overall group**	**NSR cohort**	**AF cohort**	**p-value**
**n**	101	66	35	-
**Male sex**	73 (72.3%)	48 (65.8%)	18 (64.3%)	1.00*
**Age, years**	58.9 ± 10.9	58.8 ± 11.1	59.1 ± 10.6	0.89
**Body surface area, m**^**2**^	2.07 ± 0.23	2.04 ± 0.22	2.11 ± 0.26	0.16
**Hypertension**	39 (38.6%)	25 (37.9%)	14 (40.0%)	0.83*
**Beta blocker**	46 (45.5%)	26 (39.4%)	20 (57.1%)	0.10*
**Digoxin**	5 (5.0%)	2 (3.0%)	3 (8.6%)	0.34*
**Anti arrhythmic agent**	39 (38.6%)	29 (43.9%)	10 (28.6%)	0.14*
**HR at time of CMR**	69 ± 17	61 ± 9	84 ± 20	<0.0001
**HR range, bpm**	37-121	37-76	44-121	
**SBP, mmHg**	129 ± 16	132 ± 16	124 ± 15	0.02
**DBP, mmHg**	73 ± 11	73 ± 10	74 ± 13	0.45
**LVEF, %**	56.4 ± 11.1	59.5 ± 9.4	49.7 ± 11.6	<0.0001
**LVEDVI, ml/m**^**2**^	76.7 ± 15.4	78.1 ± 15.9	72.7 ± 13.7	0.16
**LVESVI, ml/m**^**2**^	33.5 ± 13.5	32.3 ± 13.7	37.0 ± 12.6	0.17
**LA diameter, cm**	3.9 ± 0.9	3.7 ± 0.7	4.4 ± 0.9	0.0001

*Fisher’s exact test.

[Key: SBP- systolic blood pressure; DBP- diastolic blood pressure; LVEF- left ventricular ejection fraction; LVEDVI- left ventricular end-diastolic volume indexed to body surface area; LVESVI- left ventricular end-systolic volume indexed to body surface area; LA- left atrium].

### Image quality

**Figure 4:**
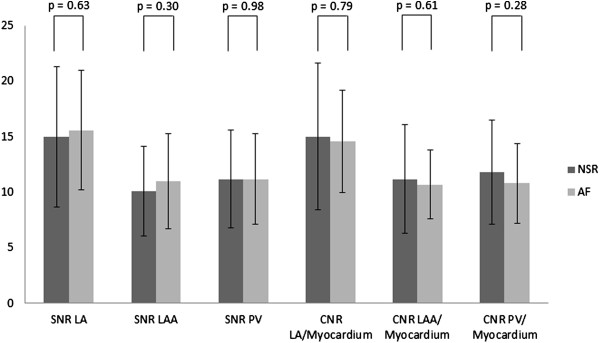
Comparison of signal to noise ratios (SNRs) and contrast to noise ratios (CNRs) between patient cohorts in normal sinus rhythm (NSR) versus atrial fibrillation (AF).

The mean ± SD acquisition time and acceptance rate were 7:51 ± 2:58 minutes and 54.4 ± 11.8%, respectively. The mean ± SD dose of gadobenate dimeglumine delivered was 29.8 ± 8.5 mls, over a mean ± SD infusion duration of 1:39 ± 0:28 minutes. 100 (99%) patients’ studies were considered diagnostic (consensus quality score > 1), and 91 (90.1%) were of good or excellent quality. Overall, mean ± SD consensus quality score was 3.40 ± 0.69. Among patients in NSR and AF, there were no significant differences in mean acquisition times, acceptance rates, quality scores, SNRs or CNRs, Table 2 and Figure 4.

**Table 2:** Quantitative and qualitative CMR measures presented for entire patient cohort and by AF status

	**Overall group**	**NSR cohort**	**AF cohort**	p-value^*^
**n**	101	66	35	-
**Mean ± SD acquisition time, mins**	7:51 ± 2:58	8:14 ± 3:10	7:08 ± 2:25	0.08
**Mean ± SD acceptance rate**	54.4 ± 11.8%	53.8 ± 12.3%	55.5 ± 10.8%	0.50
**Mean ± SD dose of MultiHance (mL)**	29.8 ± 8.5	29.0 ± 7.8	31.4 ± 9.7	0.18
**Mean ± SD consensus quality score**	3.4 ± 0.7	3.4 ± 0.7	3.3 ± 0.7	0.58
**Quality, n (%)**				0.58^#^
** Uninterpretable**	1 (1.0%)	0 (0%)	1 (2.9%)	
** Poor**	9 (8.9%)	8 (12.1%)	1 (2.9%)	
** Good**	40 (39.6%)	22 (33.3%)	18 (51.4%)	
** Excellent**	51 (50.5%)	36 (54.6%)	15 (42.8%)	

^*^Two tailed Student t test, unless otherwise specified; ^#^Mantel Haenszel Chi Square test.

The mean ± SD quality scores for reader 1 and 2 were 3.34 ± 0.70 and 3.31 ± 0.73, respectively (pooled t test p value = 0.57); the overall inter observer agreement for dichotomized quality scores assigned by these readers was substantial [30], (k = 0.68; 95% CI: 0.46, 0.90).

### Impact of heart rate and heart rhythm on image quality

Heart rhythm was not significantly associated with dichotomized consensus image quality (crude OR = 0.44, 95% CI: 0.08, 2.19; p = 0.49). By logistic regression, neither HR adjusting for rhythm [OR = 1.03, 95% CI = 0.98,1.09; p = 0.22] nor rhythm adjusting for HR [OR = 1.25, 95% CI = 0.20, 7.69; p = 0.81] demonstrated significant association with dichotomized image quality. Further adjusting for LA diameter, LVEF, and SBP did not significantly alter results. Similarly, models fitted to allow for effect modification or non-linear associations with HR did not yield different results. Multiple linear regression models demonstrated that SNRs and CNRs were largely independent of categorical HR after adjusting for heart rhythm and vice versa; with the exceptions of SNR_PV_ and CNR_PV/Myocardium_ which demonstrate a negative association with HR < 55 bpm compared to a reference HR category (55 < HR ≤ 90 bpm), Table 3. Although mean CNR_LA/myocardium_ was higher than mean values of both CNR_PV/myocardium_ (p < 0.0001) and CNR_LAA/myocardium_ (p < 0.0001), mean values of CNR_PV/myocardium_ and CNR_LAA/myocardium_ were similar, (p = 0.51).

**Table 3:** Association of heart rate with signal to noise and contrast to noise ratios

	**Heart rate ≤ 55 bpm (n = 21)**			**Heart rate > 90 bpm (n = 14)**		
	**β coefficient**	**95% CI**	**p value**	**β coefficient**	**95% CI**	**p value**
**SNR left atrium**	−2.32	−5.30, 0.66	0.13	0.21	−3.93, 4.34	0.92
**SNR LAA**	−1.41	−3.62, 0.80	0.21	−0.33	−3.29, 2.63	0.83
**SNR PV**	−2.29	−4.42,-0.17	0.03	−1.06	−4.01, 1.88	0.48
**CNR LA/Myocardium**	−2.19	−5.16, 0.78	0.15	−0.23	−4.35, 3.90	0.91
**CNR LAA/Myocardium**	−1.77	−4.12, 0.58	0.14	−0.52	−3.67, 2.63	0.75
**CNR PV/Myocardium**	−2.26	−4.38,-0.14	0.04	−1.10	−4.04, 1.85	0.46

Legend: Beta coefficients from multiple linear regression models with the respective SNR or CNR as the dependent variable, and heart rhythm and categorical HR as predictor variables. Results for HR categories presented in this table are compared to the following ‘reference’ HR category: 55 < HR ≤ 90 bpm (n = 66), and adjusted for heart rhythm.

### Image segmentation quality

97 (96%) patients’ MRA datasets were segmented using CartoMerge semiautomated image integration software. Segmentation quality scores were good or excellent for 75 (77.3%) patients, with a mean ± SD score of 2.91 (+/− 0.63). There were no significant differences in segmentation quality scores between NSR and AF patient cohorts, Table 4.

**Table 4:** Segmentation quality scores for entire patient cohort and by AF status

	**Overall group**	**NSR cohort**	**AF cohort**	**p-value**
**n**	**97**	**63**	**34**	**-**
**Mean** ± **SD segmentation score**	2.91 ± 0.63	2.92 ± 0.58	2.88 ± 0.73	0.86*
**Segmentation quality score, n (%)**				0.78^#^
Poor	1 (1.0%)	0 (0%)	1 (2.9%)	
Moderate	21 (21.7%)	13 (20.6%)	8 (23.5%)	
Good	61 (62.9%)	42 (66.7%)	19 (55.9%)	
Excellent	14 (14.4%)	8 (12.7%)	6 (17.7%)	

*Student's t-test; ^#^Mantel-Haenszel Chi-Square test.

### Patient outcomes

94 of 101 (93.1%) patients included in this study proceeded to catheter ablation of AF. Three complications occurred in 2 (2.1%) patients: right phrenic nerve injury and heart block requiring permanent pacemaker insertion occurred at time of procedure in the same patient, and atrioesophageal fistula presented in another patient 17 days after ablation. Follow up data for recurrent arrhythmia were available for 91 of 94 (96.8%); 76 (83.5%) remained in NSR after median (IQR) follow up of 308 (87, 385) days following ablation. The median (IQR) interval from ablation to recurrence of atrial arrhythmias in 15 patients (16.5%) was 348 (191, 414) days.

## Discussion

In our study, we found that free breathing 3D respiratory and ECG gated gadolinium enhanced MRA of the LA and PVs on a 3 T system is both feasible and reproducible, achieving diagnostic images in almost all patients (99%) and good or excellent diagnostic quality images in 90% of patients. Image quality is independent of HR or heart rhythm at the time of imaging. Furthermore, the quality of image segmentation obtained from these 3D MRA datasets using image integration software of an electroanatomic mapping system is high.

Fast heart rates and irregular rhythm, common among patients undergoing pre ablation LA and PV imaging, can compromise quality and increase radiation dose of gated CT imaging [31,32]. However, image quality achieved with this MRA technique is independent of HR or rhythm at the time of imaging. Thus, this MRA technique is particularly suited for ECG gated imaging of this patient cohort. The likely explanation for this independent association between image quality and either HR or rhythm is that acquisitions were gated to coincide with relatively quiescent ventricular end-systole. At higher heart rates, the reduction in end-systolic duration is less than the reduction in diastolic duration [24], rendering this phase of the cardiac cycle less sensitive to faster rates and arrhythmia. This is the largest report of respiratory gated MRA of LA and PVs acquired during ventricular end-systole, and the robust quality achieved during this phase of the cardiac cycle, despite HR or rhythm, raises the suggestion that ventricular end-systole may be a suitable target for ECG gating during late gadolinium enhancement (LGE) CMR sequences for detection of atrial fibrosis in select patients with lower heart rates that provide sufficient opportunity for required inversion times. Studies reporting left atrial scarring on 3D LGE CMR are restricted to the mid-diastolic window [33,34], and comparative studies of alternative strategies would be informative.

Although still practical, the overall mean acquisition time for this 3D respiratory and ECG gated MRA sequence of 7:51 ± 2:58 minutes is longer than acquisition times reported for commonly employed contrast enhanced MRA in expiratory breath hold, without ECG gating (5:45 ± 1:53 minutes) [13]. Furthermore, although the acquisition window is usually shorter for end-systolic compared to mid-diastolic imaging, mean acquisition time in this study was shorter than that reported in two smaller studies of 3D respiratory and ECG gated MRA of pulmonary veins where mid-diastolic ECG gating was used [13,16]. This increase in acquisition times compared to breath held, ungated MRAs may, in part, be offset by reductions in cardiac motion artifact associated through ECG gating and higher spatial resolution. While this study establishes the feasibility and high quality of 3D respiratory and ECG gated MRA, direct comparison with conventional breath held, ungated MRA was not performed in this study, and so conclusions about superiority of one technique over another cannot be made. Whether this technique reduces registration errors during image integration into electroanatomic mapping systems requires investigation.

The 3D dataset produced by free breathing respiratory and ECG gated MRA offers the potential to obtain a range of important data using a single test prior to catheter ablation of AF:

(i) High image quality facilitates PV anatomy delineation.

(ii) Using post processing software, the 3D dataset facilitates accurate measurements of PV diameters with orthogonal planes and the double oblique technique.

(iii) LA volume can be quantified using 3D chamber reconstruction technique using similar software; LA volumes have been shown to predict AF recurrence post ablation [35,36].(iv) Anatomical relationship of the pulmonary veins to esophagus and descending thoracic aorta can be reviewed. A simple and quick axial T1 weighted, fat saturated 3D gradient echo sequence is helpful in demonstrating these anatomical relationships in our experience (Figure 1).

(v) High quality PV segmentation using image integration software of an electroanatomic mapping system as shown in this study, facilitating intra procedural image guidance.

(vi) The SNRs and CNRs for the LA and LAA were similar in this study. Contrast opacification of the LA and LAA is generally good with this technique. No LA or LAA thrombi were identified within this patient cohort. Further studies to determine if intra atrial or appendage thrombus can be reliably detected using this technique, with comparisons to transesophageal echocardiography, would be interesting.

Furthermore, these data can be provided for a wide range of patients as HR, heart rhythm and ability to breath hold do not render patients ineligible for this imaging technique. These data could be further supplemented with an estimation of LV systolic function by acquiring additional images at the time of CMR; LV systolic dysfunction is another predictor of post ablation AF recurrence [37]. Similarly, to increase the diagnostic yield from gadolinium administration, quantification of atrial and PV antral fibrosis related to either AF or previous ablation procedures using high spatial resolution 3D LGE CMR could be performed at the same examination; such fibrosis is associated with LAA thrombus formation, AF recurrence post ablation and clinical outcomes [33,38-40]. There is the potential to provide a comprehensive pre-procedure report with this range of complementary data derived from this technique that could optimize electrophysiologists’ assessment of likelihood of sustained procedural success as well as procedural risk.

### Study limitations

Direct comparison of this 3D respiratory and ECG gated MRA sequence for imaging pulmonary veins to conventional contrast enhanced, breath held, ungated MRA is necessary, and is a major limitation of this study. While this study establishes the feasibility and high quality of 3D respiratory and ECG gated MRA for imaging pulmonary veins, the lack of a direct comparison precludes conclusions regarding superiority of one technique over another. Superiority over breath-held, ungated MRA will need to be investigated in future studies in order to justify longer acquisition times associated with 3D respiratory and ECG gated MRA. In addition, direct comparison to CTA would be informative. Such comparisons should include an assessment of registration errors during image integration into electroanatomic mapping systems. Certain patients, such as those with claustrophobia, may not tolerate longer acquisition times, but patient tolerance may be improved by the free breathing nature of this respiratory gated technique. Changes in LA and PV anatomy that occur during breath held imaging techniques may predispose to registration errors during image integration into electroanatomic mapping systems [15]; whether imaging in the free breathing state, in its similarity to the breathing pattern during electroanatomic mapping at catheter ablation, offers any advantage in terms of improvement in clinical outcomes is uncertain.

## Conclusions

Pre ablation imaging of the PVs and LA by end systolic 3D respiratory and ECG gated gadolinium enhanced MRA on a 3 T system is a feasible technique that achieves high quality images, reproducible image interpretation, and high quality image segmentation when imported into electroanatomic mapping systems. Image quality is independent of HR and heart rhythm for this free breathing, radiation free strategy. This technique offers an alternative strategy for pre-ablation imaging of PVs and LA to current CMR or CT based approaches, and comparative effectiveness studies are necessary to determine the optimal approach.

## Abbreviations

3D: Three dimensional; 3 T: Three tesla; AF: Atrial fibrillation; CI: Confidence interval; CMR: Cardiovascular magnetic resonance imaging; CNR: Contrast to noise ratio; CT: Computerized tomography; CTA: CT angiography; ECG: Electrocardiogram; FA: Flip angle; FOV: Field of view; HR: Heart rate; LA: Left atrium; LAA: Left atrial appendage; LGE: Late gadolinium enhancement; LVEF: Left ventricular ejection fraction; MR: Magnetic resonance; MRA: Magnetic resonance angiography; MRI: Magnetic resonance imaging; NSR: Normal sinus rhythm; OR: Odds ratio; PV: Pulmonary vein; ROI: Region of interest; SBP: Systolic blood pressure; SD: Standard deviation; SNR: Signal to noise ratio; SSFP: Steady state free precession; TE: Echo time; TR: Repetition time; TI: Inversion time; TSA: Total surface area.

## Competing interests

The authors declare that they have no competing interests.

## Authors’ contributions

All authors (JDG, AHW, TSV, GFM, MFDC, RB, RYK, MS) made substantial contributions to the conception, design, drafting, and critical revision of the manuscript. All authors read and approved the final manuscript.
